# Design and fabrication of a microstrip triplexer with wide flat channels for multi-band 5G applications

**DOI:** 10.1371/journal.pone.0302634

**Published:** 2024-05-08

**Authors:** Salah I. Yahya, Farid Zubir, Mohammed Abdel Hafez, Lewis Nkenyereye, Muhammad Akmal Chaudhary, Maher Assaad, Leila Nouri, Abbas Rezaei, Noorlindawaty Md Jizat

**Affiliations:** 1 Department of Communication and Computer Engineering, Cihan University-Erbil, Erbil, Iraq; 2 Faculty of Electrical Engineering, Wireless Communication Centre, Universiti Teknologi Malaysia, Johor Bahru, Johor, Malaysia; 3 Department of Electrical and Communication Engineering, United Arab Emirates University, Al Ain, United Arab Emirates; 4 Department of Computer & Information Security, Sejong University, Seoul, Korea; 5 Department of Electrical and Computer Engineering, College of Engineering and Information Technology, Ajman University, Ajman, United Arab Emirates; 6 Institute of Research and Development, Duy Tan University, Da Nang, Vietnam; 7 School of Engineering & Technology, Duy Tan University, Da Nang, Vietnam; 8 Department of Electrical Engineering, Kermanshah University of Technology, Kermanshah, Iran; 9 Faculty of Engineering, Multimedia University, Persiaran Multimedia, Cyberjaya, Selangor, Malaysia; UAE University, UNITED ARAB EMIRATES

## Abstract

In this paper, a new microstrip triplexer is designed to work at 2.5 GHz, 4.4 GHz and 6 GHz for mid-band 5G applications. All channels are flat with three low group delays (GDs) of 0.84 ns, 0.75 ns and 0.49 ns, respectively. Compared to the previously reported works, the proposed triplexer has the minimum group delay. The designed triplexer has 18.2%, 13.7%, 23.6% fractional bandwidths (FBW%) at 2.5 GHz, 4.4 GHz and 6 GHz, respectively. The obtained insertion losses (ILs) are low at all channels. These features are obtained without a noticeable increase in the overall size. A novel and simple resonator is used to design the proposed triplexer, which includes two pairs of coupled lines combined with a shunt stub. A perfect mathematical analysis is performed to find the resonator behavior and the layout optimization. The type of shunt stub is determined mathematically. Also, the smallness or largeness of some important physical dimensions is determined using the proposed mathematical analysis. Finally, the designed triplexer is fabricated and measured, where the measurement results verify the simulations.

## Introduction

Microstrip planar passive filtering devices with high performance have been demanded by modern wireless communication systems [1–7]. A microstrip triplexer is a type of RF filtering devices that combines three different frequency bands into a single output port. It is commonly used in communication systems to separate and combine signals in different frequency ranges. A microstrip triplexer typically consists of three bandpass filters designed to pass specific frequency bands while attenuating unwanted frequencies. Meanwhile, several microstrip triplexers using different resonators have been designed and presented in [8–24]. The multiple-mode resonators in [8] and step-impedance cells in [9] are used. In [10], a new impedance matching circuit (but with a large size) is proposed to integrate three bandpass filters (BPFs). To design a triplexer, the hairpin and interdigital microstrip cells are used in [11]. The microstrip triplexers in [11–14] occupy a large implementation area. Coupled U-shape resonators are utilized to design a microstrip triplexer in [15]. A new configuration of the coupled step impedance resonators is presented in [16] to obtain a triplexer for Worldwide Interoperability for Microwave Access (WiMAX) applications. The proposed triplexer in [17] is designed using coupled zigzag lines for GSM and Wireless Local Area Network (WLAN) applications. The presented triplexers in [16–19] have the problem of large sizes. The designers in [20,21] could reduce their triplexers sizes significantly. However, these triplexers have narrow channels and high insertion losses. All of the reported microstrip triplexers in [8–24] (except the design in reference [10]), have narrow channels with low fractional bandwidths (FBWs). Another common problem in the previous works is the high insertion losses. The distortions created by a high group delay can cause some problems. Accordingly, group delay (GD) is an important parameter in the design of microstrip devices. However, due to the difficulty of obtaining a high-performance microstrip triplexer with low GD, the referred triplexers in this work did not pay attention to this issue.

In this paper, a new small microstrip triplexer with low insertion loss, low group delay and wide FBWs is presented for 5G mid-band applications that cover from 1 GHz up to 6 GHz. The design improvements in this triplexer can have significant benefits for 5G applications in terms of signal integrity, data throughput, and compatibility with multi-band communication systems. The improvements, such as optimized impedance matching, reduced insertion loss, and improved group delay, can enhance signal integrity in 5G applications. This leads to better signal quality, reduced signal distortion, and improved overall system performance. These advancements contribute to the overall performance and reliability of 5G networks, supporting the growing demand for high-speed, low-latency connectivity in modern communication systems. The proposed resonator includes two pairs of coupled lines and a low-impedance shunt stub cell. Using the mathematical analysis of the proposed resonator, we concluded that the internal stub must be a low-impedance cell. The Z-matrix and insertion loss of the proposed resonator is calculated. Then, three BPFs are designed and integrated using the analyzed resonator. Finally, to show the features of this work we will compare it with the previously reported designs.

## Design and structure analysis

### A) Mathematical analysis of the proposed basic resonator

**Fig 1:**
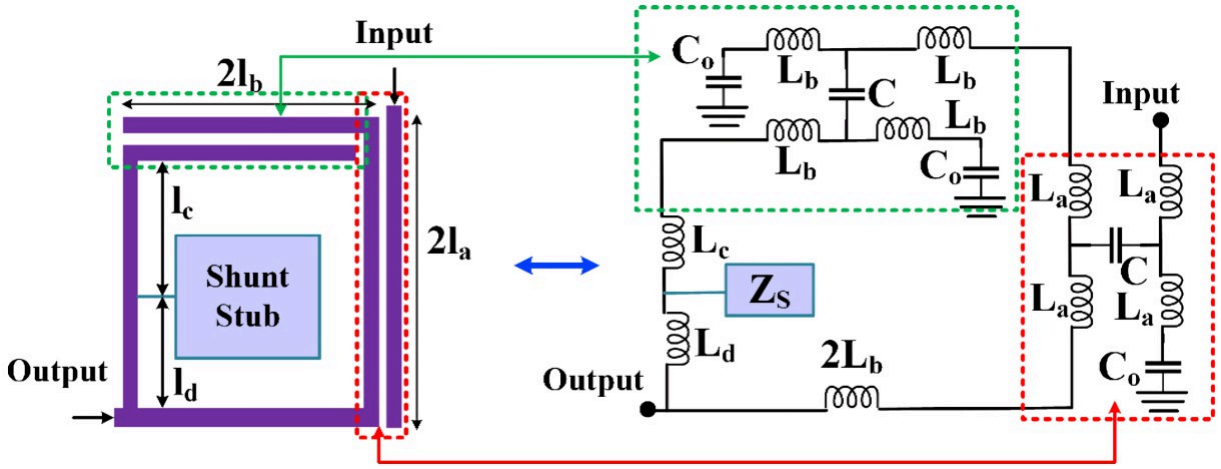
Physical structure of the proposed resonator with its approximated *LC* circuit, where the equivalents of l_a_, l_b_, l_c_, l_d_, coupling and open-end capacitors are L_a_, L_b_, L_C_, L_d_, C and C_O_ respectively.

The coupled lines have a significant effect on creating bandpass channels. Accordingly, our proposed resonator includes two pairs of coupled lines with a shunt internal stub as depicted in Fig 1. Also, an approximated LC circuit of the proposed resonator is shown in Fig 1. This equivalent of coupled lines is not exact. However, it is very effective in analyzing the proposed resonator. In the exact model of coupled lines, we have to increase the number of coupling capacitors (*C*). Moreover, we ignored the equivalent of the bents in our approximated LC model. Because they are usually significant at high frequencies (>10 GHz). The equivalent of the physical lengths l_a_, l_b_, l_c_ and l_d_ are the inductors L_a_, L_b_, L_c_ and L_d_ respectively. The open ends of coupled lines are replaced by C_O_ capacitors, while the shunt stub impedance is presented by Z_S_.

**Fig 2:**
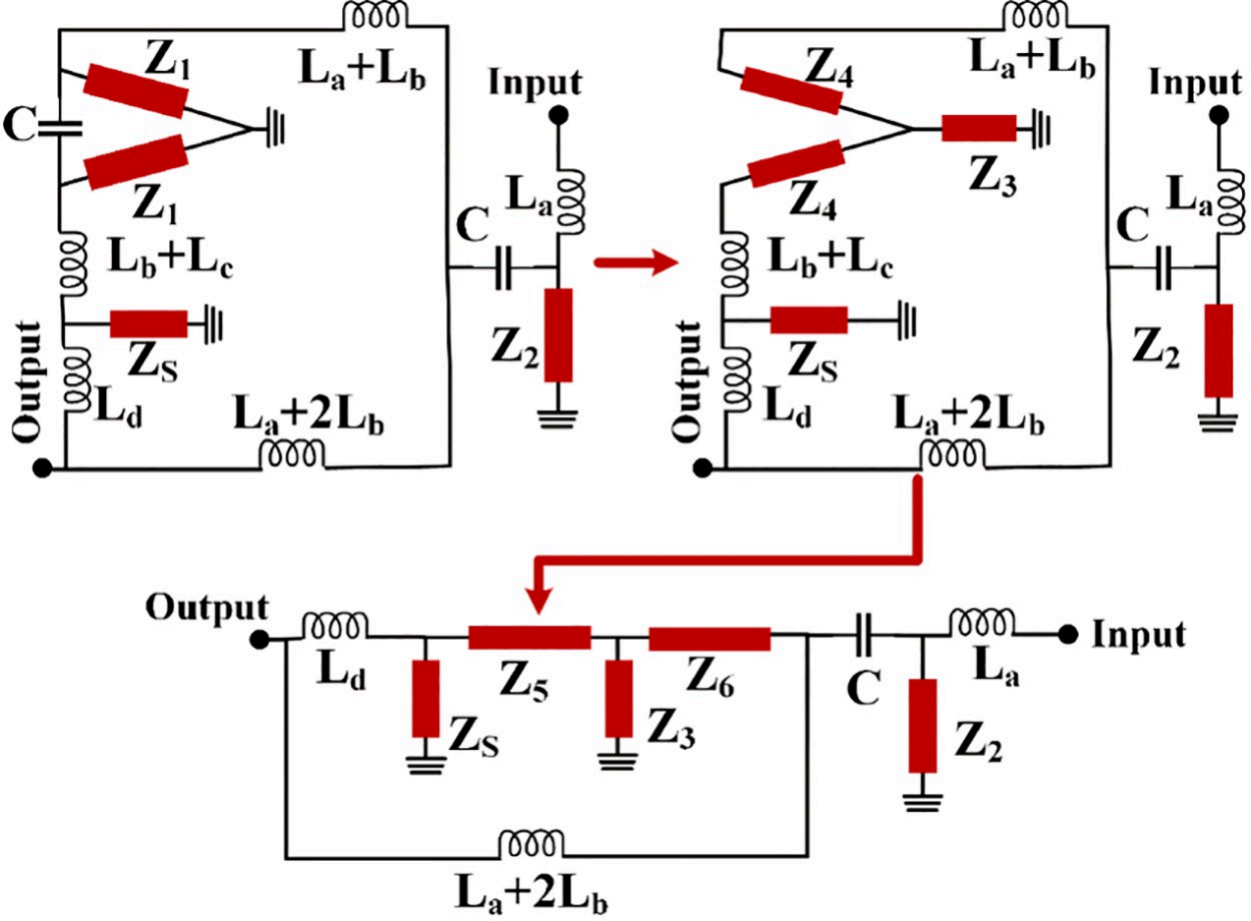
Simplifying the LC circuit (using Δ-Y transformation).

Fig 2 illustrates the steps of simplifying the proposed *LC* circuit, while the values of the impedances *Z*_*1*_ and *Z*_*2*_ are:

$$Z_{1}=j\omega L_{b}+\frac{1}{j\omega C_{O}}\;\;\&\;\;Z_{2}=j\omega L_{a}+\frac{1}{j\omega C_{O}}$$ (1)

Where ω is an angular frequency, which can be tuned at a desired point. Using *Z*_*1*_, the values of the impedances *Z*_*3*_ and *Z*_*4*_ can be calculated according to the Δ-Y transformation as follows:

Z=3Z122Z1+1jωC⇒Z=3(jωLb+1jωCO)22(jωLb+1jωCO)+1jωCZ=41jωCZ12Z1+1jωC⇒Z=4Z12Z1jωC+1⇒Z=41−ω2LbCO2jωC(1−ω2LbCO)+jωCO (2)

Usually, the coupling capacitors have small values in fF [25]. On the other hand, we want to have the inductors in nH ranges. Therefore, for a predetermined resonance frequency in GHz the values of *Z*_*3*_ and *Z*_*4*_ will be near zero and *Z*_*1*_, respectively. Using Z_4_, the values of the impedances Z_5_ and Z_6_ can be obtained by:

$$\begin{array}{l} Z_{5}=Z_{4}+j\omega (L_{c}+L_{b})\Rightarrow Z_{5}=\omega L_{b}+\frac{1}{j\omega C_{O}}\;+j\omega (L_{c}+L_{b})=\frac{1}{j\omega C_{O}}\;+j\omega (L_{c}+2L_{b})\\ Z_{6}=Z_{4}+j\omega (L_{a}+L_{b})\Rightarrow Z_{6}=\omega L_{b}+\frac{1}{j\omega C_{O}}\;+j\omega (L_{a}+L_{b})=\frac{1}{j\omega C_{O}}\;+j\omega (L_{a}+2L_{b}) \end{array}$$ (3)

By calculating the impedances values, the Z-matrix of our proposed two-port network (*Z*_*R*_) is given by:

$$Z_{R}=\left[\begin{array}{l} Z_{11\;}\;\;\;Z_{12}\\ Z_{21}\;\;\;\;\;Z_{22} \end{array}\right]$$ (4)

Where

$$\begin{array}{l} Z_{11}=j\omega L_{a}+Z_{2}\;\;\;\;\\ Z_{21}=Z_{12}=Z_{2} \end{array}$$ (5)

Since *Z*_*3*_ is near zero, by replacing a short circuit instead of it, the value of *Z*_*22*_ can be defined as follows:

$$Z_{22}=\frac{Z_{7}Z_{8}}{Z_{7}+Z_{8}}\;$$ (6)

Where

$$\left\{\begin{array}{ll} Z_{7} & =j\omega L_{d}+\frac{Z_{S}Z_{5}}{Z_{S}+Z_{5}}\;\\ Z_{8} & =\frac{(Z_{2}+\frac{1}{j\omega C})Z_{6}}{(Z_{2}+\frac{1}{j\omega C})+Z_{6}}+j\omega (L_{a}+L_{b})\Rightarrow Z_{8}\approx Z_{6}+j\omega (L_{a}+L_{b})=\frac{1}{j\omega C_{O}}\;+j\omega (2L_{a}+3L_{b}) \end{array}\right.$$ (7)

From the above equations and for a small value of coupling capacitor, we can obtain the impedance Z_7_ as follows:

$$\begin{array}{l} Z_{7}=j\omega L_{d}+\frac{Z_{S}(\frac{1-\omega^{2}L_{b}C_{O}}{2j\omega C(1-\omega^{2}L_{b}C_{O})+j\omega C_{O}}+j\omega (L_{c}+L_{b}))}{Z_{S}+\frac{1-\omega^{2}L_{b}C_{O}}{2j\omega C(1-\omega^{2}L_{b}C_{O})+j\omega C_{O}}+j\omega (L_{c}+L_{b})}\Rightarrow \\ Z_{7}\approx j\omega L_{d}+\frac{Z_{S}(\frac{1-\omega^{2}L_{b}C_{O}}{j\omega C_{O}}+j\omega (L_{c}+L_{b}))}{Z_{S}+\frac{1-\omega^{2}L_{b}C_{O}}{j\omega C_{O}}+j\omega (L_{c}+L_{b})} \end{array}$$ (8)

If we replace a low-impedance section instead of *Z*_*S*_, the value of *Z*_*7*_ will be simplified:$Z_{7}\approx j\omega L_{d}$. Using the Z-parameters we can extract S_21_ parameter (transmission parameter) of the proposed resonator as follows [26]:

$$S_{21}=\frac{2Z_{21}}{(Z_{11}+Z_{0})(Z_{22}+Z_{0})-Z_{12}Z_{21}}\Rightarrow S_{21}=\frac{2Z_{0}Z_{2}}{(j\omega L_{a}+Z_{2}+Z_{0})(\frac{Z_{7}Z_{8}}{Z_{7}+Z_{8}}+Z_{0})-Z_{2}^{2}}$$ (9)

Where *Z*_*0*_ is the impedance of the terminal. By substituting *Z*_*2*_, *Z*_*7*_, and *Z*_*8*_ in Eq (9):

$$S_{21}=\frac{2Z_{0}[j\omega L_{a}+\frac{1}{j\omega C_{O}}]}{(2j\omega L_{a}+\frac{1}{j\omega C_{O}}+Z_{0})(\frac{Z_{7}Z_{8}}{Z_{7}+Z_{8}}+Z_{0})-{\left[j\omega L_{a}+\frac{1}{j\omega C_{O}}\right]}^{2}}$$ (10)

Where

$$\frac{Z_{7}Z_{8}}{Z_{7}+Z_{8}}\approx \frac{j\omega L_{d}[\frac{1}{j\omega C_{O}}\;+j\omega (2L_{a}+3L_{b})]}{j\omega L_{d}+[\frac{1}{j\omega C_{O}}\;+j\omega (2L_{a}+3L_{b})]}$$ (11)

If *L*_*d*_<<*2L*_*a*_*+3L*_*b*_ (for a short line of l_d_), then S_21_ will be changed as follows:

$$S_{21}=\frac{2Z_{0}(j\omega L_{a}+\frac{1}{j\omega C_{O}})}{(2j\omega L_{a}+\frac{1}{j\omega C_{O}}+Z_{0})(j\omega L_{d}+Z_{0})-{\left(j\omega L_{a}+\frac{1}{j\omega C_{O}}\right)}^{2}}$$ (12)

From the above equations, we can calculate the insertion loss (IL) and the conditions to have an ideal IL (near zero) as follows:

$$\begin{array}{l} IL=-20\;\text{log}\;\left|\frac{2(j\omega L_{a}+\frac{1}{j\omega C_{O}})Z_{0}}{(2j\omega L_{a}+\frac{1}{j\omega C_{O}}+Z_{0})(j\omega L_{d}+Z_{0})-{\left(j\omega L_{a}+\frac{1}{j\omega C_{O}}\right)}^{2}}\right|\;\;(dB)\\ IL\approx 0\Rightarrow 2(j\omega L_{a}+\frac{1}{j\omega C_{O}})Z_{0}=(2j\omega L_{a}+\frac{1}{j\omega C_{O}}+Z_{0})(j\omega L_{d}+Z_{0})-{\left(j\omega L_{a}+\frac{1}{j\omega C_{O}}\right)}^{2} \end{array}$$ (13)

If we assume that, the line with the physical length l_d_ is very small, then for the small value of *L*_*d*_ Eq (13) will be simplified as follows:

$$\begin{array}{l} 2(j\omega L_{a}+\frac{1}{j\omega C_{O}})Z_{0}=(2j\omega L_{a}+\frac{2}{j\omega C_{O}})(Z_{0})+Z_{0}^{2}-{\left(j\omega L_{a}+\frac{1}{j\omega C_{O}}\right)}^{2}-\frac{Z_{0}}{j\omega C_{O}}\Rightarrow \\ Z_{0}^{2}+\omega^{2}L_{a}^{2}+\frac{1}{\omega^{2}C_{O}^{2}}-\frac{2L_{a}}{C_{O}}-\frac{Z_{0}}{j\omega C_{O}}=0\Rightarrow \left\{\begin{array}{l} Z_{0}^{2}+\omega^{2}L_{a}^{2}+\frac{1}{\omega^{2}C_{O}^{2}}-\frac{2L_{a}}{C_{O}}=0\\ \frac{Z_{0}}{j\omega C_{O}}\approx 0 \end{array}\right. \end{array}$$ (14)

For the predetermined values of angular frequency and *Z*_*0*_, the ratio of *L*_*a*_*/C*_*O*_ can be obtained. Using this method, we can tune the resonance frequency and decrease the insertion loss simultaneously. As mentioned before, the physical length l_d_ should be short and the internal shunt stub should be a low-impedance section.

### B) Design of BPFs using the analyzed resonator

**Fig 3:**
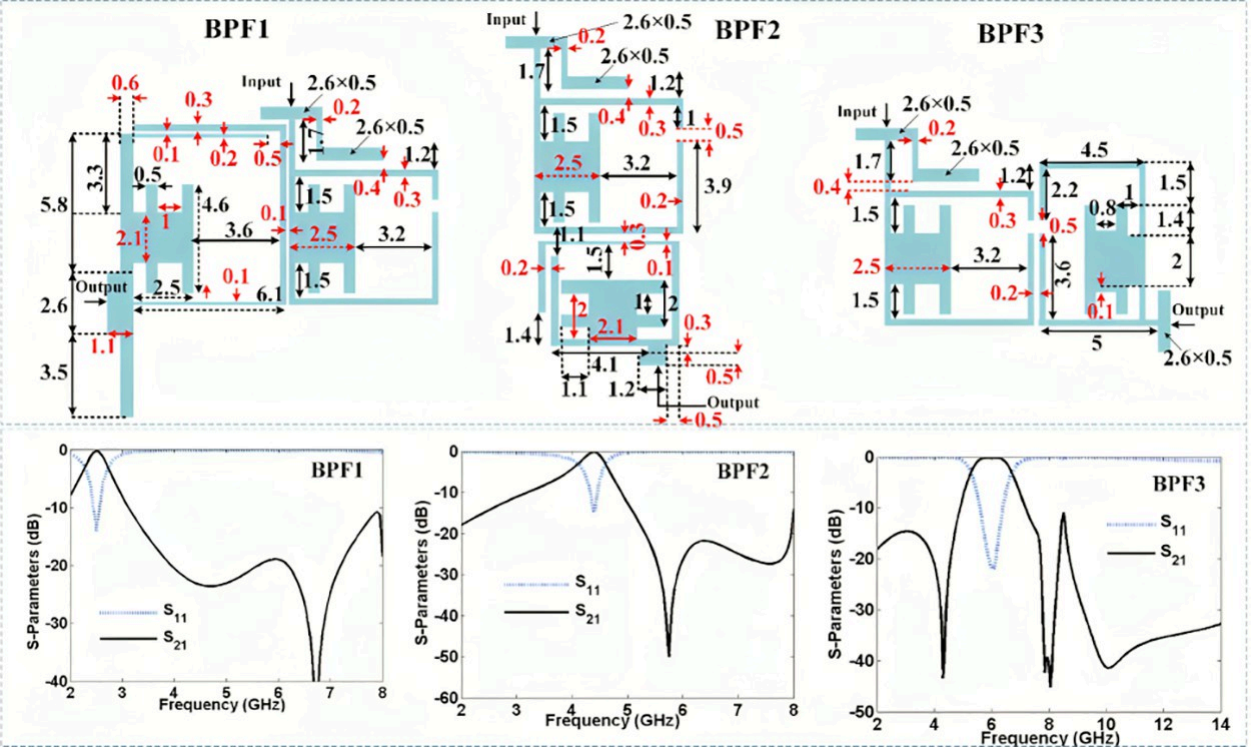
The proposed BPFs with their frequency responses, where all dimensions are written in mm.

Using this mathematical analysis, we could achieve the resonator behavior to tune the dimensions and the layout optimization. Based on the analyzed resonator, three BPFs (BPF1, BPF2, and BPF3) are designed and simulated using ADS software on a Rogers RT/duroid 5880 substrate with tan(δ) = 0.0009, h = 0.7874 mm and 2.22 dielectric constant. The linear steps of EM simulator are used. The use of a Rogers RT/Duroid 5880 substrate with low dielectric constant, low loss tangent, and high reliability has contributed to the development of high-performance microstrip triplexer, enabling improved signal processing and communication in various RF applications. Fig 3 depicts the proposed BPFs with their frequency responses (all dimensions are in mm). The simulation results show that BPF1, BPF2 and BPF3 work at 2.5 GHz, 4.4 GHz and 6 GHz respectively. Moreover, their simulated insertion losses are better than 0.3 dB, 0.2 dB and 0.1 dB respectively.

### C) Design of a triplexer using the proposed BPFs

**Fig 4:**
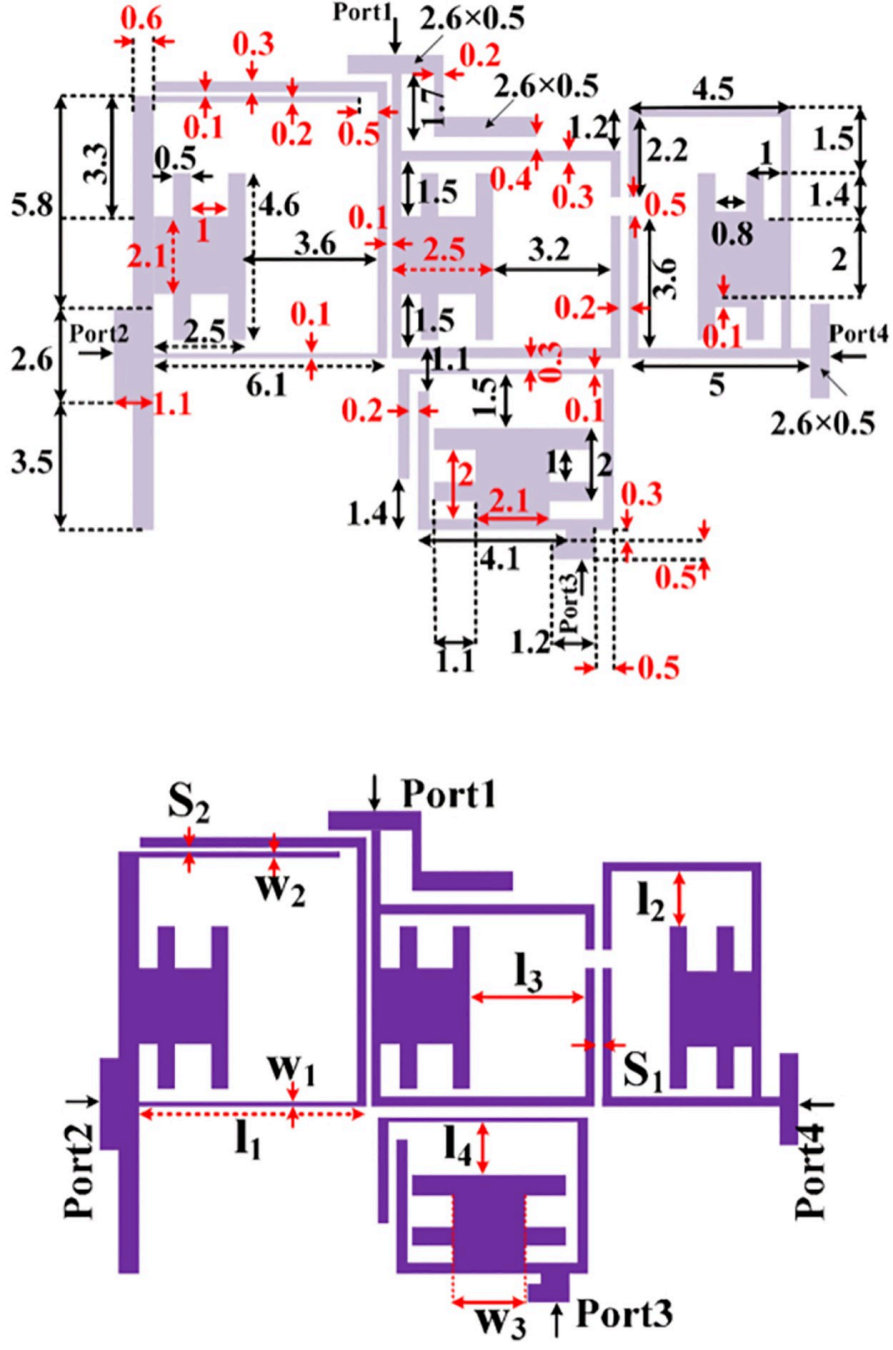
The proposed triplexer composed of BPF1, BPF2 and BPF3 (unit: mm).

**Fig 5:**
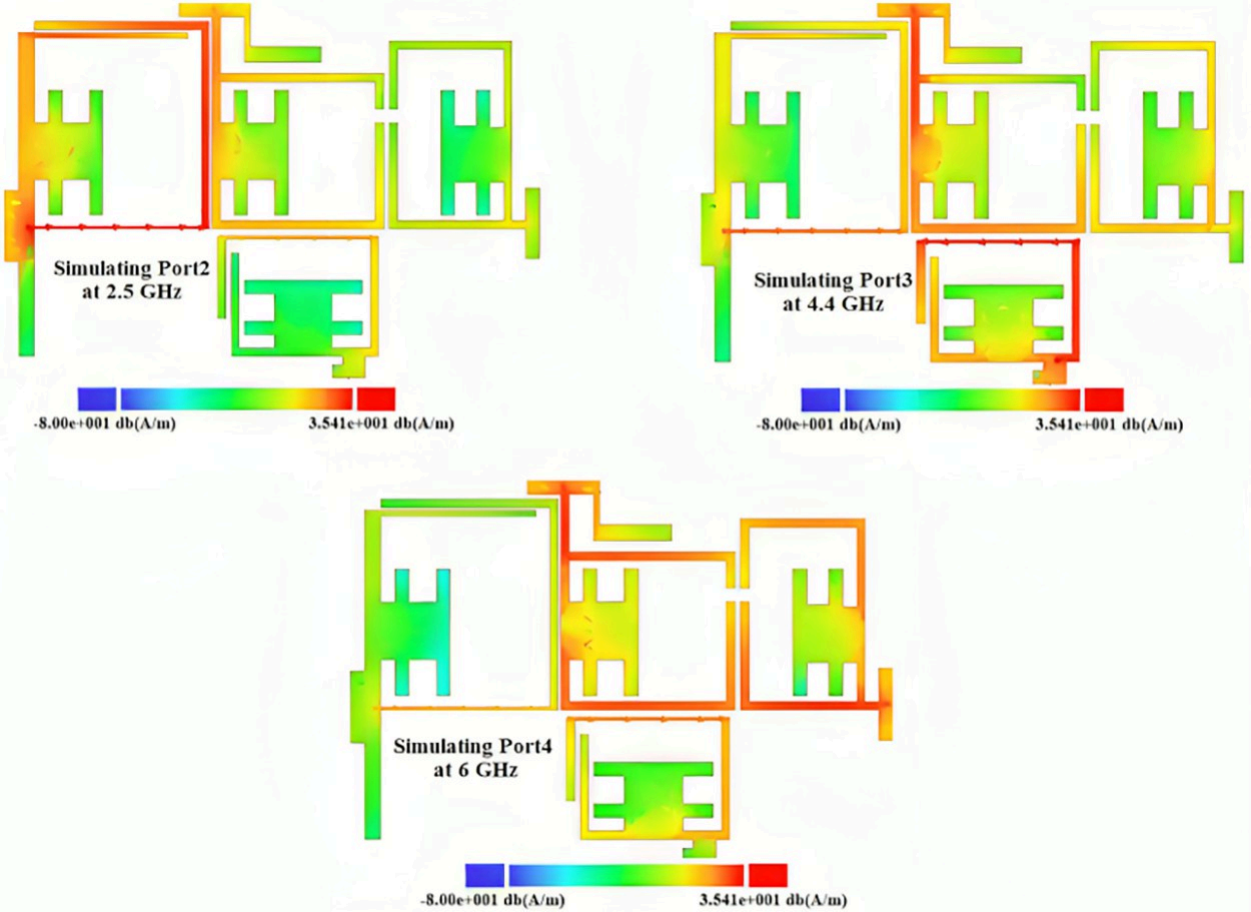
Current density distributions of the designed triplexer for simulating ports 2, 3 and 4 at 2.5 GHz, 4.4 GHz and 6 GHz, respectively.

By integrating the proposed BPFs, a low-loss triplexer is designed and presented in Fig 4. The dimensions of the proposed triplexer are the same as the BPFs dimensions. The overall size of our triplexer is 13.8 mm × 19.6 mm = 0.15λ_g_ × 0.21λ_g_, where λ_g_ is the guided wavelength calculated at the 1^st^ resonance frequency. The effective physical dimensions on the frequency response are l_1_, l_2_, l_3_, l_4_, S_1_, S_2_, w_1_, w_2_, and w_3_. We selected them based on the resonator behavior obtained from the presented mathematical analysis and the current density distributions shown in Fig 5. As shown in Fig 5, the thin lines have more current density distributions. For port 2 at 2.5 GHz, the coupled lines near this port have high current density. Meanwhile, for port 3 at 4.4 GHz the lower loop has more current density. However, this loop has a loading effect on BPF3. As can be seen in Fig 5, the coupled lines near port 4 have high current density distributions when we simulate this port at 6 GHz.

**Fig 6:**
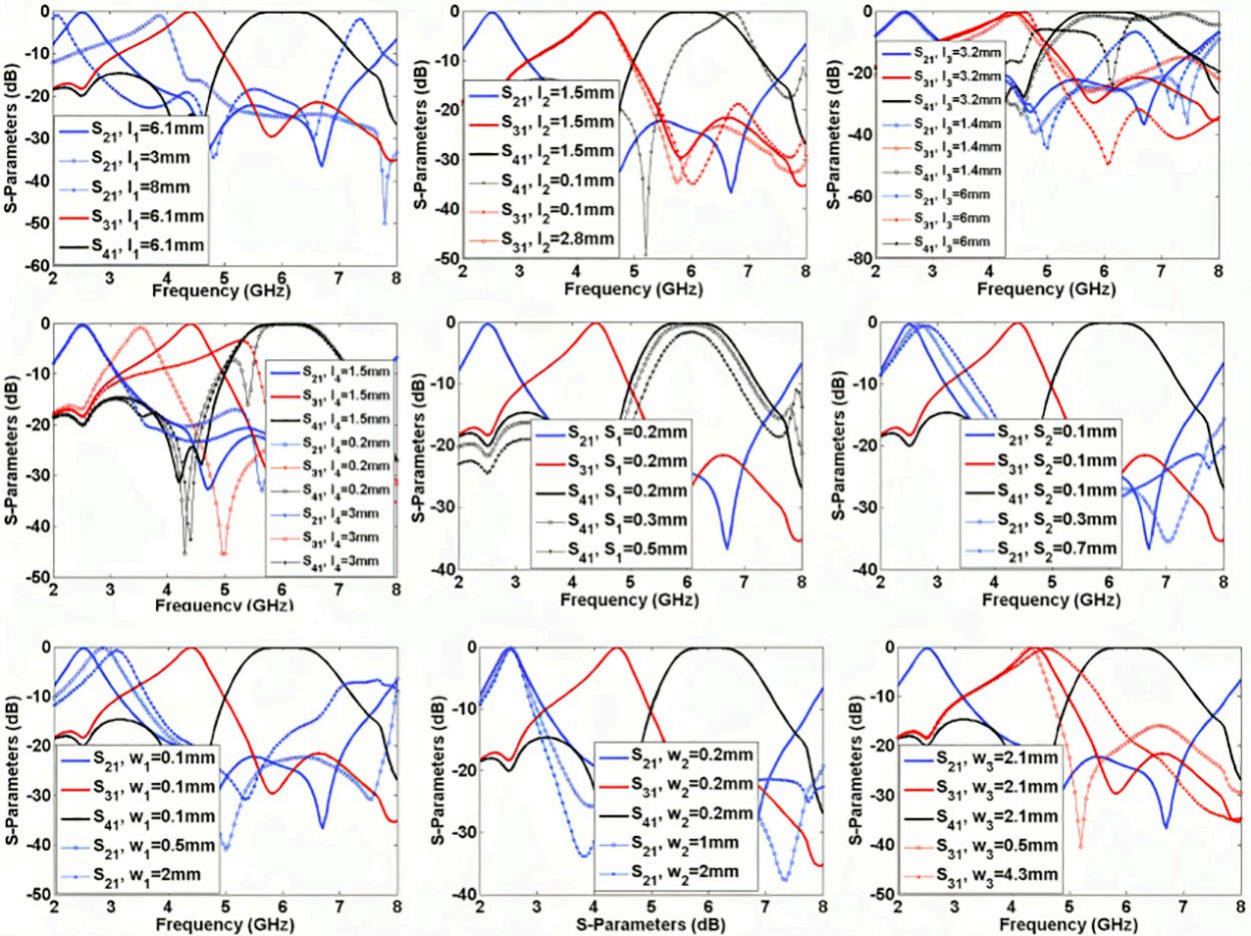
Frequency responses as nine functions of the effective physical dimensions l_1_, l_2_, l_3_, l_4_, S_1_, S_2_, w_1_, w_2_, w_3_.

**Fig 7:**
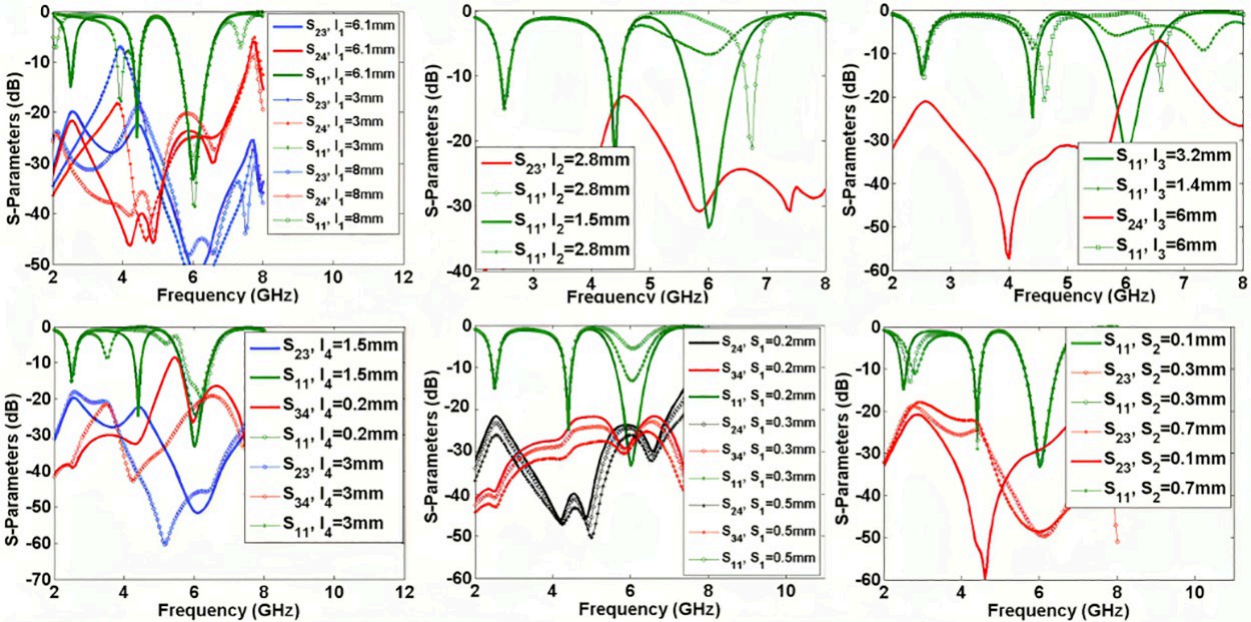
Common port return loss and isolations as six functions of the effective physical dimensions l_1_, l_2_, l_3_, l_4_, S_1_ and S_2_.

The significant physical dimensions are changed to find their effects on the frequency responses. Fig 6 shows S_21_, S_31_, and S_41_ as functions of l_1_, l_2_, l_3_, l_4_, S_1_, S_2_, w_1_, w_2_, and w_3_. As can be seen, increasing l_1_ shifts the resonance frequency of S_21_ to the left but it creates a harmonic near the 3^rd^ resonance frequency. Changing l_2_ affects the bandwidth and flatness of the 3^rd^ channel, while changing l_3_ affectsall channels. Since l_4_ is near port 3, increasing this length moves the middle channel to the left. As presented in Fig 5, increasing the space between the coupled lines (S_1_ and S_2_) can increase the insertion loss. Also, to control the created harmonics inside the last channel, we can tune the widths w_1_, w_2_, and w_3_. Fig 7 shows the effects of significant lengths and widths on the isolations and return loss. By tuning these parameters, we can control the isolation between the output ports and return loss simultaneously. As shown in Fig 7, the best values of l_1_, l_2_, l_3_, and l_4_ are 6.1 mm, 1.5 mm, 3.2 mm and 1.5 mm, respectively.

## Simulation and measurement results

**Fig 8:**
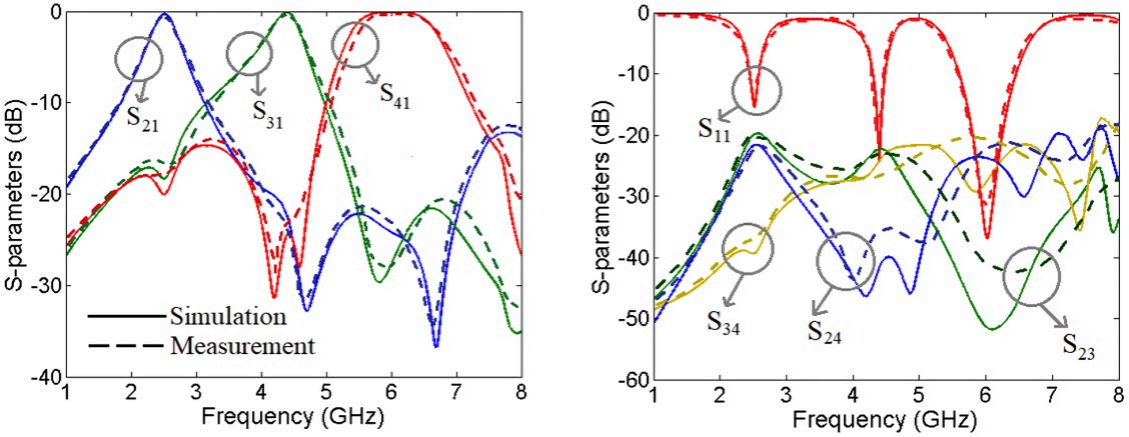
Simulated (solid lines) and measured (dashed lines) frequency responses of the proposed triplexer.

**Fig 9:**
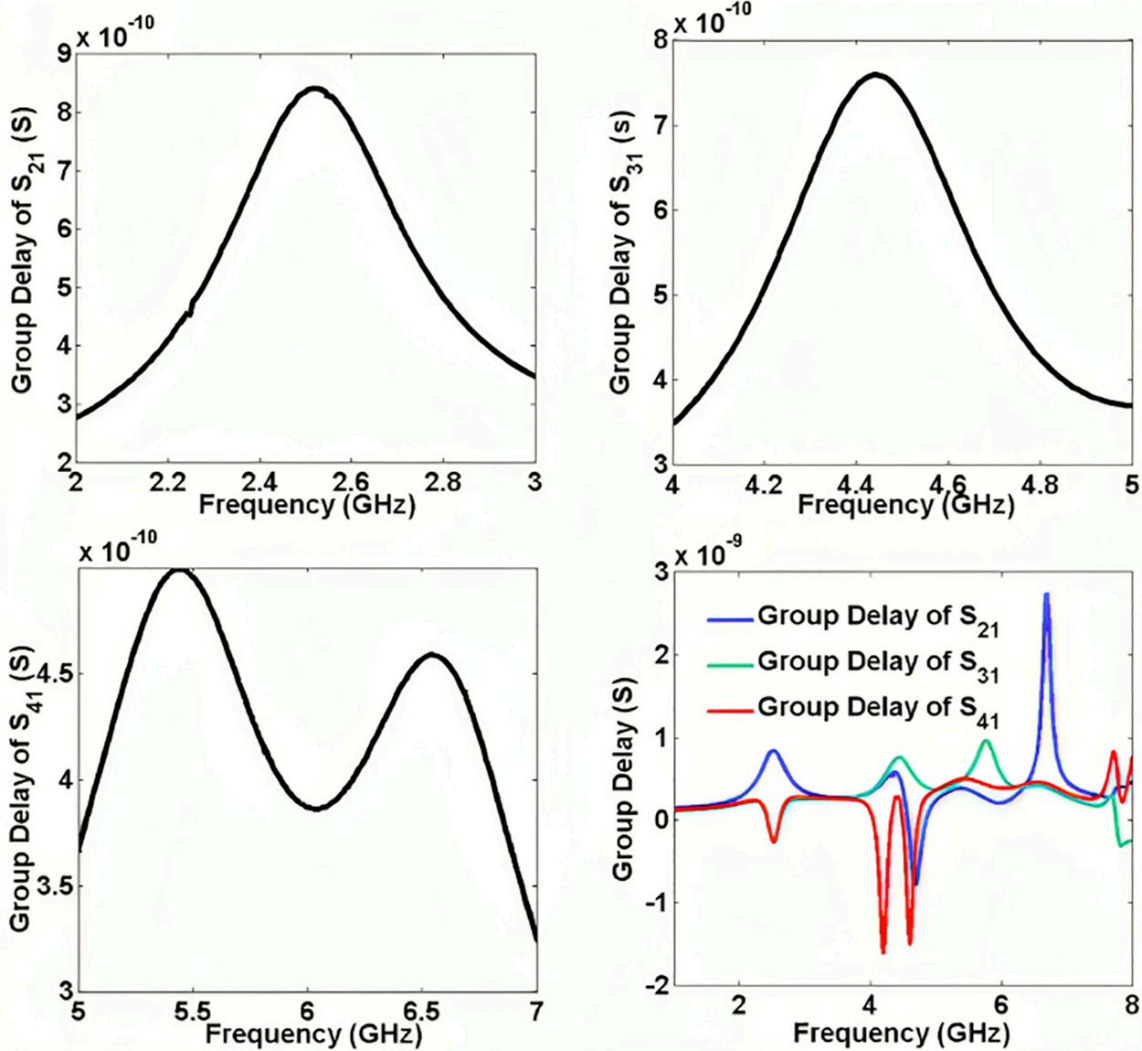
Narrowband and wideband group delays at all channels.

**Fig 10:**
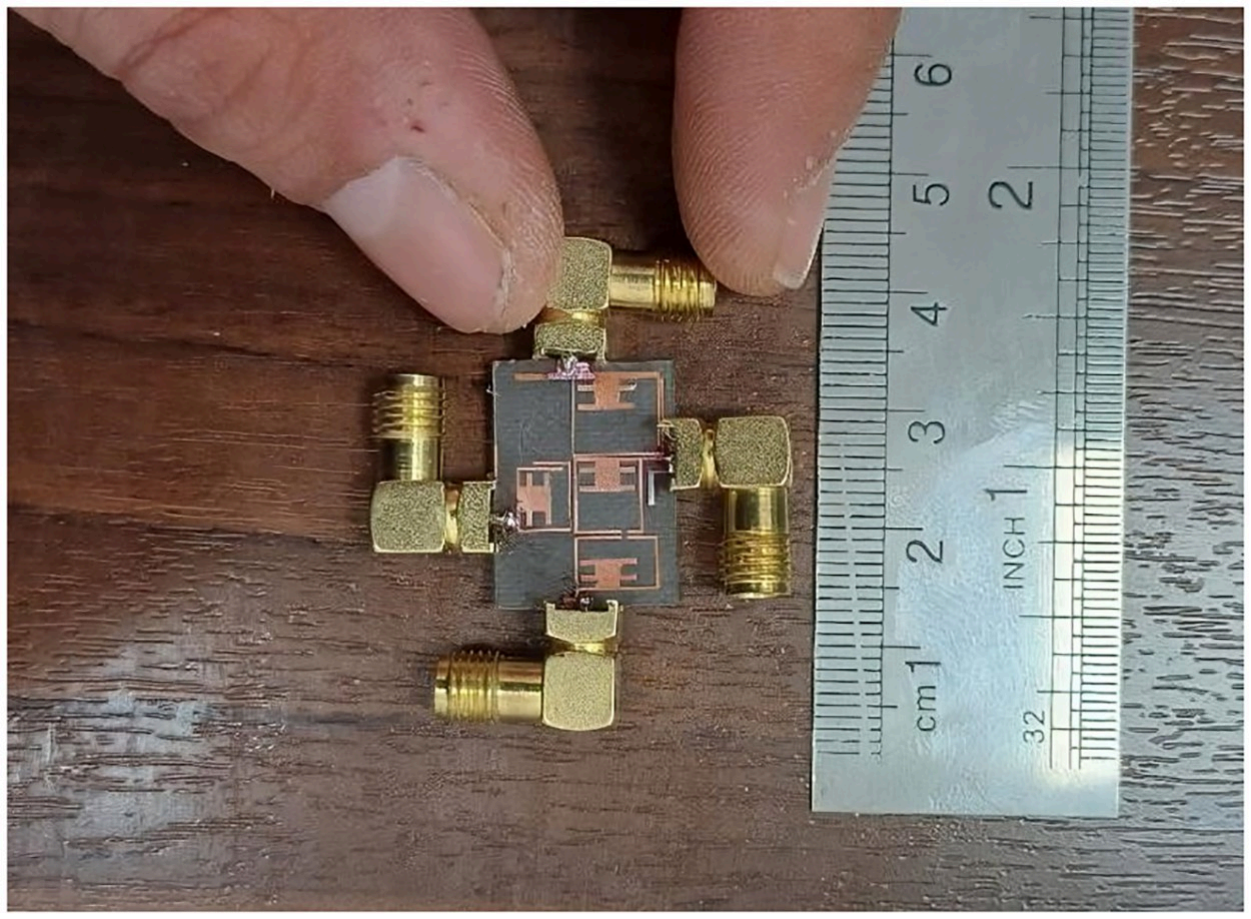
Fabricated triplexer.

The designed triplexer is simulated by the EM simulator of ADS software with a linear step of 5 MHz. Then it is fabricated and measured. The measurements are performed by an HP8757A network analyzer. Since the structure is simple, the manufacturing error is low. Hence, the simulation and measurement results are in good agreement. However, due to the copper and SMA connectors losses the simulated results are a little better than the measurement results. Variations in the dimensions of the microstrip components such as line widths, gaps, and lengths, can lead to changes in the frequency response. Also, imperfections in the surface finish of the microstrip traces can cause additional losses and alter the triplexer characteristics. Meanwhile, misalignment during the fabrication process can result in impedance mismatches and affect the triplexer response. Improper calibration of the measurement equipment can lead to inaccuracies in the measured results. Also, changes in temperature, humidity, and other environmental conditions can influence the measurement results. Therefore, the measurements must be conducted in a controlled environment to minimize the impact of external factors on the results. Fig 8 presents the simulated and measured frequency response of our triplexer. The results show that the proposed triplexer works at 2.5 GHz, 4.4 GHz, and 6 GHz, where the insertion losses at these frequencies are 0.27 dB, 0.12 dB and 0.09 dB respectively. Meanwhile, it has three common port return losses of 15.2 dB, 22.2 dB and 35.8 dB at the 1^st^, 2^nd^ and 3^rd^ channels respectively. The lower -3 dB passband is from 2.28 GHz up to 2.735 GHz. Also, the middle passband is from 4.07 GHz up to 4.635 GHz. Finally, the -3 dB cut-off frequencies of the upper channel are 5.33 GHz and 6.75 GHz. The maximum isolation between the output ports is -19.75 dB. As shown in Fig 8, the first, middle and last channels are wide and flat with 18.2%, 13.7%, 23.6% FBWs respectively. The measured insertion losses (for all channels) are about 0.5 dB higher than the simulation results. Also, the measured return loss at the 1^st^, 2^nd^ and 3^rd^ channels are near 13.9 dB, 20.5 dB and 31.2 dB respectively. These measured results verify the designing process and the simulation results with a good accuracy. The group delays of each channel inside the -3 dB bandwidth and the group delay of all channels from 1 GHz up to 8 GHz are shown in Fig 9. The results show that the group delays of the 1^st^, 2^nd^ and 3^rd^ channels are better than 0.84 ns, 0.75 ns and 0.49 ns respectively. Fig 10 shows a photograph of the fabricated triplexer.

## Comparison with the previous works

To show the advantages of our triplexer we compared its size and performance with the previous triplexers in Table 1. In this Table, the index 1, 2 and 3 are related to the 1^st^, 2^nd^ and 3^rd^ channels respectively. Also, F_O_, IL, RL and FBW are the operating frequency, insertion loss, return loss and fractional bandwidth respectively. According to the summarized comparison results, we could obtain the lowest insertion losses at all channels, the lowest common port return loss at the last channel and the best fractional bandwidths. Only the size of the proposed triplexers in [20,21] are smaller than ours. However, they have narrow channels with high insertion losses. As depicted in Table 1, usually simple structures have larger dimensions. A number of triplexers with simple structures are proposed in [8,14,15,18,19,22,24]. However, with the exception of [15] all of them have overall sizes greater than 0.1 λg^2^. Despite being simple, the proposed structure in this work is the smallest compared to the previous works which is a special advantage.

**Table 1:** Comparison between our triplexer and the previous works (*: Approximated values).

Refs.	F_O1_, F_O2_, F_O3_ (GHz)	IL1, IL2, IL3 (dB)	RL1, RL2,RL3 (dB)	FBW1, FBW2, FBW3	Layout Complexity	Size (λg^2^)
This Triplexer	2.5, 4.4, 6	0.27, 0.12, 0.09	15.2, 22.2, 35.8	18.2%, 13.7%, 23.6%	Low	0.03
[8]	3.2, 3.7, 4.4	2.7, 2.5, 1.8	16, 16, 16	6.5%, 7%, 8%	Middle	0.048
[9]	1.4, 1.7, 1.9	3.4, 3.5, 3.6	—	4.96%, 4.57%, 4.82%	Low	0.358
[10]	1.2, 1.8, 2.4	1.3, 1.3, 1.2	11.6, 14, 10	14.4%, 14%, 13.6%	High	0.055
[11]	3.3, 3.89, 4.56	2.2, 2.3, 2.3	Better than 14	—	High	0.275
[12]	0.9, 2.45, 5.35	0.37, 0.68, 0.4	11.8, 21.3, 13.8	—	High	0.088
[13]	1.5, 1.7, 1.9	4.9, 5.8, 5.95	—	3.3%, 2.9%, 3.6%	Middle	0.132
[14]	1.88, 2.1, 2.6	1.3, 2.3, 3.2	22, 25, 21	0.86%, 1.4%, 0.96%	Low	0.1*
[15]	1, 1.25, 1.5	2.7, 1.8, 3.2	Better than 16	9.5%, 4.2%, 4.5%	Low	0.064
[16]	2.67, 3.1, 3.43	0.72, 0.63, 0.71	24.5, 24, 24.7	—	High	0.137
[17]	0.9, 2.4, 5.5	0.7, 1.7, 1.5	—	—	Middle	—
[18]	1.8, 3.2, 4.4	1.97, 1.99, 2.3	24, 22, 25	7.44%, 7.45%, 6.2%	Low	0.177
[19]	2.4, 3.5, 5.8	0.9, 1.1, 1.3	—	6%, 4.5%, 3.6%	Low	0.119
[20]	1.75, 2.35, 3.68	1.3, 1.4, 1.7	20, 25, 30	5.7%, 8.5%, 6.8%	High	0.027
[21]	1.45, 2.15, 2.75	3.6, 4.3, 4.8	15, 20, 15	6%, 6%, 4%	High	0.020
[22]	2.4, 3.5, 5.2	2.42, 1.62, 1.95	Better than 15	3%, 7%, 3%	Low	0.164
[23]	2.3, 3.2, 3.6	0.78, 1.1, 0.62	19.8, 10, 28	5.2%, 5.5%, 1.6%	Middle	0.095
[24]	2.05, 2.45, 3.5	1.5, 1.8, 1.5	Better than 13	4.8%, 4%, 5.7%	Low	0.346

The group delay distortion can cause problems such as poor analog video and audio. Because it can change the signal waveform as it passes through the system. Accordingly, the group delay is a very significant parameter in microwave design. One way to reduce the group delay is properly matching the input and output impedance which is well done for this triplexer. This method can help to minimize reflections and improve the group delay response. Another way to have a low group delay is to increase the fractional bandwidth. The lowest group delay belongs to the second channel of this triplexer, which has the widest fractional bandwidth. The simulation results show that by increasing the dimensions of the stubs inside the loops, the fractional bandwidth can be decreased. Moreover, using high-quality substrate materials with low dielectric loss can help to reduce group delay in the passbands. However, none of the reported triplexers has investigated this parameter. Therefore, we have to compare the group delay of this work with the other microstrip passive filtering devices such as diplexers and filters. Table 2 shows a comparison between the microstrip devices in terms of group delay, types and the number of channels. Although the design of a triplexer is more complicated than the diplexers and filters, we could achieve the lowest group delay at all channels.

**Table 2:** Group delay comparison (*: Approximated values).

References	Number of Channels	Type	Group Delays (ns)
This work	3	Triplexer	0.84, 0.75, 0.49
[27]	4	Diplexer	2.76, 3.31, 0.91, 2.15
[28]	3	BPF	Better than 8nson all channels
[29]	2	Diplexer	3, 3.14
[30]	3	BPF	3.67, 1.47, 0.83
[31]	2	Diplexer	3.15, 2.98

## Conclusion

In this work, we designed a new microstrip triplexer with low insertion loss and low group delay. It has a novel and simple structure. The maximum group delay and the maximum insertion losses at all channels are 0.84 ns and 0.27 dB, respectively, while the overall size of our triplexer is only 0.03λ_g_^2^. Using the mathematical analysis of the proposed resonator, we could find its behavior that helped us to tune the dimensions. Another achievement of the mathematical analysis of the presented resonator is introducing a method to tune the resonance frequency and reduce the insertion loss simultaneously. Finally, we optimized our triplexer structure to improve its performance compared to the previous triplexers.
